# Crystal Structure of the Lamprey Variable Lymphocyte Receptor C Reveals an Unusual Feature in Its N-Terminal Capping Module

**DOI:** 10.1371/journal.pone.0085875

**Published:** 2014-01-23

**Authors:** Ryo Kanda, Yoichi Sutoh, Jun Kasamatsu, Katsumi Maenaka, Masanori Kasahara, Toyoyuki Ose

**Affiliations:** 1 Graduate School of Life Sciences, Hokkaido University, Sapporo, Japan; 2 Department of Pathology, Hokkaido University Graduate School of Medicine, Sapporo, Japan; 3 Faculty of Pharmaceutical Sciences, Hokkaido University, Sapporo, Japan; University of Luebeck, Germany

## Abstract

Jawless vertebrates represented by lampreys and hagfish use variable lymphocyte receptors (VLRs) as antigen receptors to mount adaptive immune responses. VLRs generate diversity that is comparable to immunoglobulins and T-cell receptors by a gene conversion-like mechanism, which is mediated by cytosine deaminases. Currently, three types of VLRs, VLRA, VLRB, and VLRC, have been identified in lampreys. Crystal structures of VLRA and VLRB in complex with antigens have been reported recently, but no structural information is available for VLRC. Here, we present the first crystal structure of VLRC from the Japanese lamprey (*Lethenteron japonicum*). Similar to VLRA and VLRB, VLRC forms a typical horseshoe-like solenoid structure with a variable concave surface. Strikingly, its N-terminal cap has a long loop with limited sequence variability that protrudes toward the concave surface, which is the putative antigen-binding surface. Furthermore, as predicted previously, its C-terminal cap lacks a highly variable protruding loop that plays an important role in antigen recognition by lamprey VLRA and VLRB. Recent work suggests that VLRC+ lymphocytes in jawless vertebrates might be akin to γδ T cells in jawed vertebrates. Structural features of lamprey VLRC described here suggest that it may recognize antigens in a unique manner.

## Introduction

All classes of jawed vertebrates (gnathostomes) ranging from mammals to the cartilaginous fish employ immunoglobulins (Igs) and T-cell receptors (TCRs) as antigen receptors [1]. In contrast, jawless vertebrates (cyclostomes), such as lampreys and hagfish, have neither Igs or TCRs. Evidence indicates that variable lymphocyte receptors (VLRs) play roles equivalent to those of Igs and TCRs in the adaptive immune system of cyclostomes [2], [3]. VLRs acquire their diversity through rearrangement of leucine-rich repeat (LRR) modules [4], [5], whereas the recombination of variable (V), diversity (D) and joining (J) gene segments is responsible for the generation of diversity in both B-cell receptors and TCRs [6], [7]. VLRs consist of an N-terminal cap (LRRNT), the first LRR (LRR1), multiple (usually up to seven) 24-residue variable LRRs (LRRVs), a terminal or end LRRV (LRRVe), a connecting peptide (CP) and a C-terminal cap (LRRCT), followed by an invariant 3′-terminal region [4]. Sequence diversity of mature VLRs is generated by the step-wise insertion of an LRR-encoding module into the immature, incomplete *VLR* gene during the development of lymphocytes [8], [9]. This assembly of *VLR* genes is thought to occur through a gene conversion-like mechanism mediated by cytosine deaminases of the AID-APOBEC family [10], [11].

Previously, two types of VLRs, VLRA and VLRB, were identified in lampreys and hagfish [5], [10], [12]. Recently, a third VLR, designated VLRC, was identified in lampreys [13], [14]. VLRA and VLRC are believed to be type I membrane proteins with a C-terminal transmembrane region. In contrast, VLRB is attached to the cell membrane via a glycosyl-phosphatidylinositol (GPI) anchor [4]. Like Igs, VLRB can be secreted by plasma cells as pentamers or tetramers of dimers, which function as strong agglutinins [15]. VLRA, VLRB and VLRC are expressed on distinct populations of lymphocytes [4], [13], [16], and functional *VLR* gene assembly occurs monoallelically, enabling expression of a single VLR on each lymphocyte. Interestingly, VLRA+ cells express orthologs of genes typically expressed in gnathostome T-lineage cells, whereas VLRB+ cells show gene expression patterns similar to gnathostome B-lineage cells [16], indicating that T-like and B-like lymphocyte-like cells emerged before the divergence of gnathostomes and cyclostomes. VLRC sequences are more closely related to VLRA than to VLRB sequences, leading to the speculation that VLRC+ cells are T-lineage cells and that, like jawed vertebrates equipped with two lineages of T-cells (αβ and γδ T-cells) and one lineage of B-cells, lampreys may have two lineages of T-like cells (analogous to αβ and γδ T-cells) and one lineage of B-like cells [13]. Recent work demonstrated that lamprey VLRA+ and VLRC+ cells are distinct lineages of T-like cells and that they might be functionally akin to αβ and γδ T-cells of jawed vertebrates, respectively [17].

Crystallographic analysis of hagfish VLRA and VLRB revealed that VLRs have a horseshoe-like solenoid structure typical of the LRR family of proteins, such as Toll-like receptors [18]. Subsequently, antigen recognition mechanisms of VLR were unveiled from the structures of lamprey VLRB in complex with H-trisaccharide [19], hen egg white lysozyme (HEL) [20], or the immunodominant glycoprotein of *Bacillus anthracis* spores [21]. Surprisingly, lampreys immunized with HEL produced not only specific VLRBs, but also specific VLRAs exhibiting higher affinity than VLRBs [12]. The crystal structure of lamprey VLRA in complex with HEL revealed that VLRA can recognize antigens directly [22], which was suggested to be analogous to direct recognition of the non-classical major histocompatibility complex (MHC) class I molecule T22 by the mouse γδ TCR [23], [24]. Here we present the first crystal structure of VLRC from the Japanese lamprey *Lethenteron japonicum*. Interestingly, the N-terminal cap of VLRC has a long loop with limited sequence variability that protrudes toward the concave, putative antigen-binding surface.

Very recently, Li et. al. reported the presence of a third VLR in hagfish and suggested that the newly identified VLR is the counterpart of lamprey VLRA whereas the hagfish VLR molecule previously known as VLRA is the counterpart of lamprey VLRC [25].

## Materials and Methods

### Production of the VLRC ectodomain

The DNA fragment encoding the ectodomain of VLRC including LRRNT, LRR1, LRRV1, LRRV2, LRRV3, LRRVe, CP and LRRCT (amino acid residues 25−246; accession no. AB507271) was amplified from cDNA [13] using 5′- GGGAATTCCATATGGCTTGCCTTGCGGTCGGCAAGGATGAC-3′ as a forward primer and 5′-CCGGAATTCTTAATTGCAAGTCACATTCTTGATTTTTTC-3′ as a reverse primer (underlined bases indicate restriction sites *Nde*I and *Eco*RI introduced for cloning purposes). The “stalk domain” was not included in the construct because its amino acid sequence is invariant. The resultant fragment was digested with *Nde*I and *Eco*RI, and ligated into the modified expression plasmid pET-26(b) (Novagen, USA) in fusion with an N-terminal 10× His tag. Modified pET-26(b) contained the downstream box sequence (ATGAATCATA) [26] before the start codon. The protein was overproduced in *E. coli* strain C43 (DE3). Single colonies were selected and grown overnight at 310 K in preculture media containing Luria broth with 25 µg ml^−1^ kanamycin. The precultures were then transferred into flasks containing 1 l of 2× YT medium with 25 µg ml^−1^ kanamycin. When the cell density reached an OD_600_ of 0.6, isopropyl 1-thio-β-D-galactopyranoside (IPTG) was added to the media to a final concentration of 1 mM to induce protein expression. Cells were cultured for a further 6 h at 310 K, and harvested by centrifugation at 5000× *g* for 30 min at 277 K and washed with a buffer containing 50 mM Tris-HCl (pH 8.0) and 150 mM NaCl.

Inclusion bodies were isolated from cell pellets by sonication and washed repeatedly with a wash solution containing 0.5% Triton X-100. Purified VLRC inclusion bodies were solubilized in a denaturant solution that included 6 M guanidine hydrochloride. By using the refolding buffer (0.1 M Tris-HCl (pH 8.5), 0.6 M L-arginine, 2 mM EDTA, 3.73 mM cystamine, 6.73 mM cysteamine), the solubilized protein solution was diluted slowly (2 ml min^−1^) to a final concentration of 1−2 µM and stirred for 72 h at 4°C. The solution, which included refolded VLRC, was then concentrated with a VIVAFLOW50 system (Sartorius, USA) followed by gel filtration with a HiLoad 26/60 Superdex 75 prep grade column (GE Healthcare, USA). The purity of the protein was assessed on a 15% SDS-PAGE. A sole band with a molecular mass band ∼25 kDa was observed, corresponding to the molecular mass of the VLRC ectodomain.

### Crystallization

Prior to crystallization trials, VLRC was concentrated to a final concentration of 10 mg ml^−1^ in a buffer containing 10 mM Tris-HCl (pH 8.0) and 50 mM NaCl. Concentration was carried out using a Millipore centrifugal filter device (Amicon Ultra-4, 10 kDa cutoff; Millipore, USA). Screening for crystallization was performed using Wizard Screens I and II (Emerald Biosciences, USA), JBScreen Classic 1–10 (Jena Bioscience, Germany), JCSG + suite, PACT suite, and the PEGs and PEGs II suites (Qiagen, Germany) by the sitting-drop vapor diffusion method in 96-well plates (SWISSCI MRC 2 Well, Jena Bioscience, Germany). A drop of 0.1 µl of the sample was mixed with an equal volume of reservoir solution. The mixture was equilibrated against 0.1 ml of reservoir solution at 293 K. Crystals were grown from PEGs suite #89 (0.2 M potassium phosphate, 20% (w/v) PEG3350). Based on this result, an extensive optimization in a 96-well sitting-drop format was carried out. A diffraction-quality crystal was obtained by mixing 0.2 µl protein solution (15 mg ml^−1^) and 0.2 µl reservoir solution [0.2 M potassium phosphate, 20% (w/v) PEG3350], and then equilibrating the drops against 60 µl of the reservoir solution at 293 K.

### Data collection and structure determination

X-ray diffraction data were collected at beamline NW12 of the Photon Factory Advanced Ring (PF-AR, Tsukuba, Japan) using an ADSC CCD detector Q210. Prior to diffraction data collection, crystals were cryoprotected by transfer into a solution containing 25% (v/v) glycerol for a few seconds and flash-cooled. The data set was integrated, merged and scaled using HKL-2000 [27]; the crystal diffracted up to 2.1 Å. The VLRC crystal belonged to space group *P2_1_2_1_2*, with unit-cell parameters *a*  =  102.2, *b*  =  37.2, *c*  =  55.1 Å. Based on the value of the Matthews coefficient (*V*_M_) [28], it was estimated that there was one molecule in the asymmetric unit with *V*_M_  =  2.21 A^3^/Da (*V*solv  =  44.5%). Details of the data collection and processing statistics are given in Table 1.

**Table 1:** Data statistics.

	VLRC
**Data collection**	PF-AR NW12
Wavelength (Å)	1.000
Resolution range (Å)	50–2.3 (2.38–2.30)
Space group	*P*2_1_2_1_2
Unit-cell parameters (Å)	*a* = 102.2, *b* = 37.2, *c* = 55.1
No. of observations	67913
No. of unique reflections	9798 (885)
Completeness (%)	100 (92.8)
Multiplicity	6.9 (5.7)
Averaged *I*/σ (*I*)	11.6 (4.1)
*R*_merge_ a	0.113 (0.322)
**Refinement**	
Protein atoms	1710
Water atoms	92
Resolution range (Å)	50–2.30 (2.63–2.30)
*R*_work_ b	0.181 (0.181)
*R*_free_ c	0.238 (0.273)
R. m. s. deviation	
Bond lengths (Å)	0.003
Bond angles (°)	0.76
Ramachandran plot^d^ (%)	
Favored	95.5
Outliers	0.0
**PDB entry**	3WO9

Values in the parentheses are for the highest resolution shell.

a *R*_merge_ = Σ_h_Σ_j_|<*I*>_h_−*I*_h,j_|/Σ_h_Σ_j_ *I*_h,j_, where <*I*>_h_ is the mean intensities of symmetry-equivalent reflections.

b *R*_work_ = Σ_h_|*F*_o_−*F*_c_|/Σ_h_ *F*_o_, where *F*_o_ and *F*_c_ are the observed and calculated structure factor amplitudes, respectively.

c *R*_free_ value was calculated for *R* factor, using only a test set of reflections (5% of the total) not used in the refinement.

d Ramachandran plot was evaluated using Molprobity [32].

The structure was solved by the molecular replacement method using the program Molrep [29]. The crystal structure of VLRA from hagfish (PDB ID: 2o6q) was used as a search model. The sequence identity between lamprey VLRC and hagfish VLRA is 53.9%. Structure refinement was carried out using Refmac5 [30] and Phenix [31]. The final model was refined to an *R*_free_ factor of 19.0% and an *R* factor of 21.6% with a root mean square deviation of 0.008 Å in bond length and 1.16° in bond angle for all reflections between 50 and 2.3 Å resolution. Table 1 also presents a summary of the statistics for structure refinement. The stereochemical properties of the structure were assessed by Molprobity [32] and COOT [33], and showed no residues in the outlier region of the Ramachandran plot. The final model comprises His25 to Cys247 out of a total of 320 residues of the VLRC molecule. The N-terminal residues with an extra 10× His-tag and the C-terminal residues are missing. Structure comparison with other VLR molecules was carried out using Secondary-Structure Matching (PDBeFold) [34].

### Sequence variability analysis

All VLR sequences used here were derived from previous studies [4], [5], [8], [10], [11] [13]. For analysis of LRRNT or LRRCT regions, multiple sequence alignments were carried out using ClustalW [35]. Shannon entropy (*H*) was computed on the HIV sequence database server (http://www.hiv.lanl.gov/content/sequence/ENTROPY/entropy_one.html). The Shannon entropy (H) for each aligned position was calculated by the following equation.
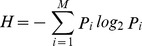
Gap openings were excluded from the calculation. The logarithm was calculated to base 2. The Shannon entropy was calculated using inshore hagfish (*Eptatretus burgeri*) VLRA or VLRB, sea lamprey (*Petromyzon marinus*) VLRA or VLRB, or Japanese lamprey VLRC. VLRC sequences with an equal number of LRRV modules were chosen for alignment of LRR modules. All multiple alignments were checked by eye and if needed, corrected to maximize the extent of similarity using MEGA 5 [36].

## Results

### Overexpression and purification of the VLRC ectodomain

Protein expression was not detected when the VLRC ectodomain (residues 25−246) construct was introduced into the pET-26(b) vector. However, high level expression of recombinant protein was obtained when a 10× His-tagged VLRC ectodomain was expressed with the modified pET-26(b) vector, in which, the downstream box sequence was inserted before the start codon. The recombinant VLRC protein was obtained as inclusion bodies, and following the refold step eluted at about the volume of the monomer species in gel filtration chromatography.

### Overall structure of the VLRC ectodomain

**Figure 1:**
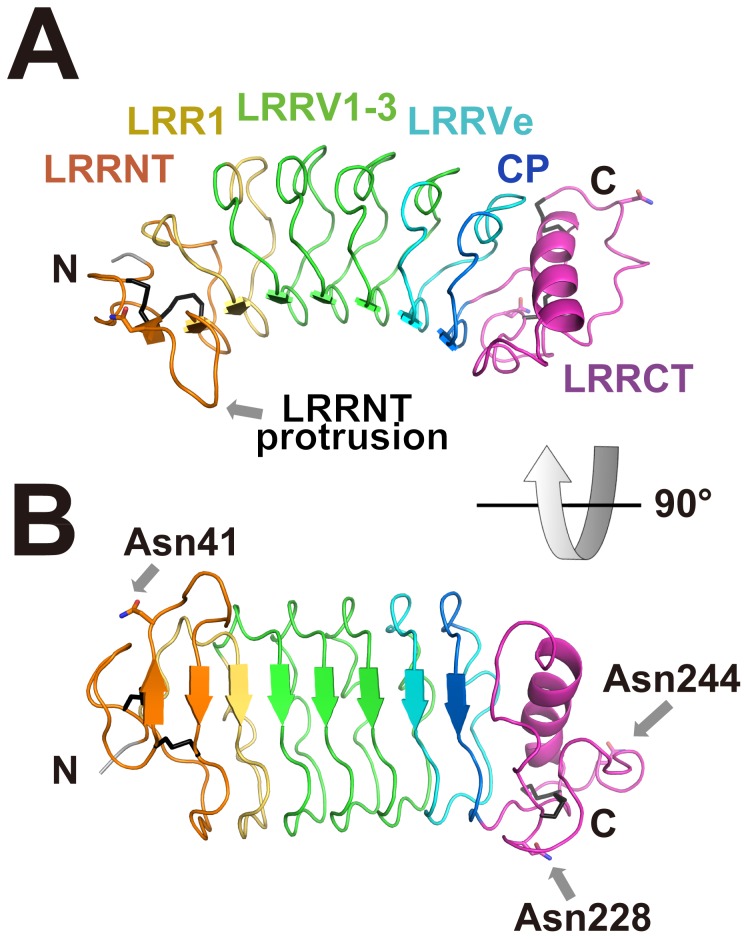
Ribbon diagram of the VLRC crystal structure. Views from the side (A) and the concave surface (B) are shown. Each region is colored as follows: LRRNT, orange; LRR1, yellow; LRRVs, green; LRRVe, cyan; CP, blue; and LRRCT, magenta. Potential *N*-linked glycosylation sites are indicated by an arrow in (B). Side chains are shown in a stick model. Disulfide bonds in LRRNT and LRRCT are shown in black in a stick model.

The crystal structure of the lamprey VLRC ectodomain (residues from His25 to Cys247) was determined by molecular replacement at a resolution of 2.3 Å. The structure has a horseshoe-like solenoid conformation, which is typical of proteins that belong to the LRR protein family (Figure 1). Lamprey VLRC is composed of eight modules, including LRRNT, LRR1, LRRV1, LRRV2, LRRV3, LRRVe, CP and LRRCT in this order. The concave surface of lamprey VLRC is formed by the β-sheet made up of eight β-strands (two from LRRNT, five from LRRs and one from CP); the β1-strand is anti-parallel whereas the remaining β-strands are arranged in a parallel fashion (Figure 1). These β-strands are the only secondary structures assigned by DSSP [37].

Lamprey VLRB molecules are secreted as disulfide bond-linked multimers with four or five pairs of protein chains [19]. The crystal structure of lamprey VLRC showed that the ectodomain of VLRC is a monomer. However, the 3′-terminal 74 residues of VLRC, of which five residues are cysteines, were not included in our crystallographic analysis. Therefore, we cannot exclude the possibility that these cysteines of VLRC facilitate the oligomerization of multimers through disulfide bond formation.

**Figure 2:**
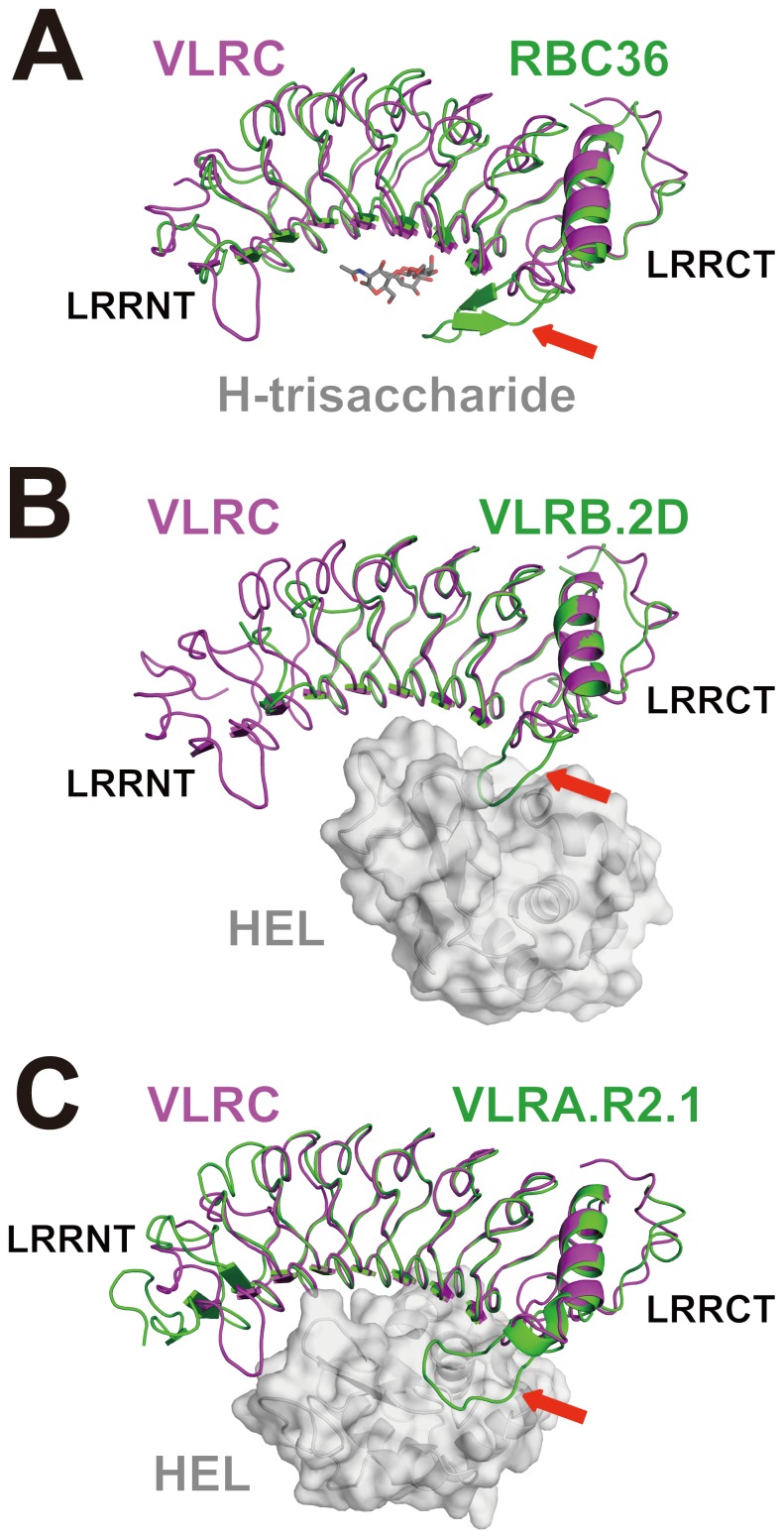
Structural comparison of VLRC with lamprey VLRA and VLRB. VLRC is shown in magenta, whereas VLRA and VLRB are shown in green and their bound ligands in gray. (A) Superimposition of VLRC and the lamprey VLRB.RBC36 trisaccharide complex [19]. (B) Superimposition of VLRC and the lamprey VLRB.2D-HEL complex [20]. (C) Superimposition of VLRC and the lamprey VLRA.R2.1-HEL complex [22]. Arrows indicate protruding loops located in the LRRCT of VLRA and VLRB.

The main chain Cα atoms from LRRNT to CP were well superimposed on the known VLR structures such as VLRB.59 (PDB ID: 2O6S, rmsd = 0.97 Å for Cα atoms of VLRC residues 35–240 and VLRB.59 residues 26–231) [18], VLRB.61 (PDB ID: 2O6R, rmsd = 1.18 Å for Cα atoms of VLRC residues 38–240 and VLRB.61 residues 29–199) [18], VLRA.29 (PDB ID: 2O6Q, rmsd = 1.23 Å for Cα atoms of VLRC residues 24–232 and VLRA.29 residues 23–239) [18], VLRA.R2.1-HEL (PDB ID: 3M18, rmsd = 1.24 Å for Cα atoms of VLRC residues 38−243 and VLRA.R2.1 residues 10–246) [22], the VLRB.RBC36 trisaccharide complex (PDB ID: 3E6J, rmsd = 1.42 Å for Cα atoms of VLRC residues 32–247 and VLR.RBC36 residues 20–238) [19], VLRB.VLR4-immunodominant glycoprotein (PDB ID: 3TWI, rmsd = 2.76 Å for Cα atoms of VLRC residues 38–242 and VLR4 residues 27–181) [21] and VLRB.2D-hen egg white lysozyme (HEL) (PDB ID: 3g3a, rmsd = 2.86 Å for Cα atoms of VLRC residues 37–247 and VLRB. 2D residues 6–166) [20] (Figure 2). Like VLRA and VLRB, N-terminal and C-terminal caps of VLRC have a total of four disulfide bonds presumably important for structural integrity: two within the LRRNT (Cys28-Cys39, Cys37-Cys52) and two within the LRRCT (Cys200-Cys227, Cys202-Cys247).

**Figure 3:**
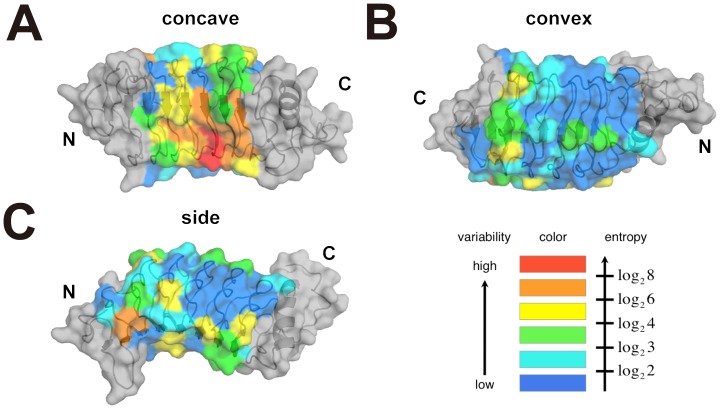
VLRC sequence diversity mapped onto the surface of the LRR and CP modules. The entropy was calculated from 75 aligned Japanese lamprey VLRC sequences. The sequence variability in LRR1, LRRV1-3, LRRVe and CP is indicated by the color gradation from red (the most variable positions: the highest entropy values) to blue (the least variable positions: the lowest entropy values). LRRNT and LRRCT are shown in gray.

**Figure 4:**
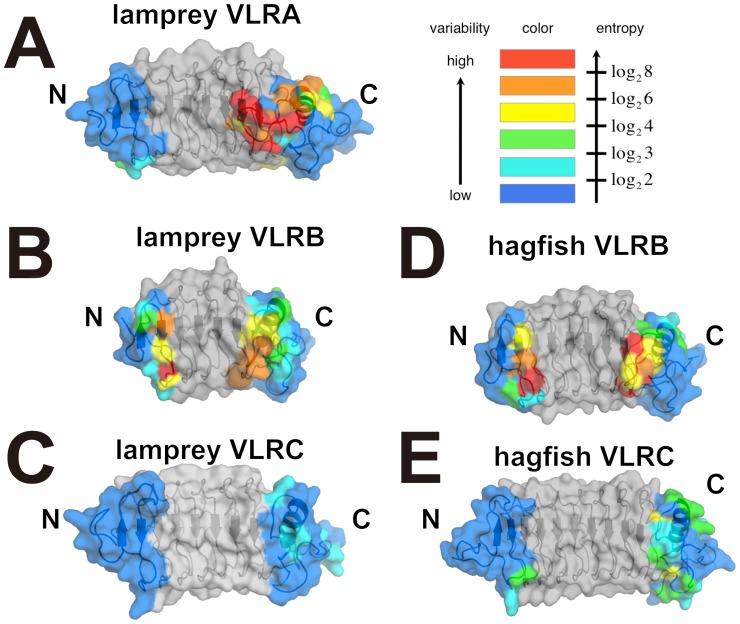
Sequence variability in LRRNT and LRRCT. The Shannon entropy was calculated from 192 sea lamprey (*Petromyzon marinus*) VLRA sequences (A), 640 sea lamprey (*Petromyzon marinus*) VLRB sequences (*B*), 102 Japanese lamprey (*Lethenteron japonicum*) VLRC sequences (*C*), 98 inshore hagfish (*Eptatretus burgeri*) VLRB sequences (*D*), or 226 inshore hagfish (*Eptatretus burgeri*) VLRC sequences (previously reported as hagfish VLRA) (*E*). The molecular surfaces were calculated with the structure of lamprey VLRA.R2.1 (PDBID 3G3A) (*A*), lamprey VLRB.2D (PDBID 3M18) (*B*), lamprey VLRC from this study (*C*), hagfish VLRB.59 (PDBID 2O6S) (*D*), and hagfish VLRA.29 (VLRC.29 according to the new nomenclature) (PDBID 2O6Q) (*E*). Sequence variability is indicated by the color gradation from red (the most variable positions) to blue (the least variable positions) as in Figure 3. LRR1, LRRVs, LRRVe and CP are shown in gray.

We calculated the Shannon entropy [20], [38], [39] for each position based on the multiple alignments of 102 Japanese lamprey VLRC sequences [13] and mapped variable residues onto the VLRC crystal structure (Figures 3 and 4). This revealed that variable residues are located predominantly on the concave surface, in particular in the region around the core β-sheet (Figure 3), suggesting that, similar to VLRA and VLRB, VLRC recognizes its ligands through the concave surface [19], [20], [21], [22] (Figure 2). In the known structures in complex with ligands, both hydrophilic and hydrophobic residues are involved in ligand recognition [19], [20], [21], [22]. The distribution of hydrophilic and hydrophobic residues on the concave surface of our crystal structure suggests that this is also the case with VLRC.

The ectodomain of lamprey VLRC contains three potential *N*-glycosylation sites, one in the LRRNT (Asn41) and two in the LRRCT (Asn228, and Asn244). However, glycosylation at these residues is unlikely to affect ligand recognition, because these residues are located outside of the concave region (Figure 1, panel B).

### A protruding loop in the N-terminal cap

**Figure 5:**
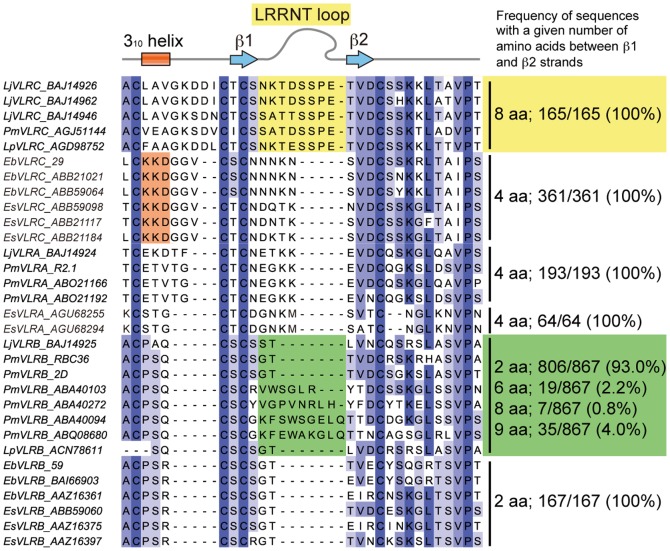
Amino acid sequences of LRRNT in known VLR molecules. Representative VLR sequences from lampreys and hagfish were aligned, and the number of residues between β-strands, β1 and β2, was counted. Frequency of sequences with a given number of residues between β1 and β2 (shown on the right side of the figure) was calculated using publicly available sequences. Note that all known lamprey VLRC sequences have eight residues between β1 and β2 (highlighted in yellow). The number of corresponding residues is four in VLRA, and >90% of VLRB sequences have two residues (highlighted in green). In hagfish VLRC, amino acid residues which form 3_10_-helices are highlighted in orange. VLR sequences are from sea lamprey (Pm, *Petromyzon marinus*), Japanese lamprey (Lj, *Lethenteron japonicum*), brook lamprey (Lp, *Lampetra planeri*), inshore hagfish (Eb, *Eptatretus burgeri*), and Pacific hagfish (Es, *Eptatretus stoutii*). VLR molecules were named according to the new nomenclature. Thus, EbVLRC and EsVLRC correspond to the previously reported EbVLRA and EsVLRA molecules, respectively. EsVLRA represents the newly described VLR [25].

A striking feature of lamprey VLRC is that its N-terminal cap has a long loop protruding toward the concave surface (Figure 1). This protrusion is formed by amino acid residues 41–48 located in the region connecting two β-strands β1 and β2 (Figure 5). These eight residues show only low sequence variability (Figure 4, panel E). Indeed, ∼80% of known VLRC sequences from *Lethenteron japonicum* have the same sequence, NKTDSSPE, and the remaining ∼20% have closely related sequences (Table 2). Essentially the same observation was made with the VLRC sequences from two other lamprey species, *Petromyzon marinus* and *Lampetra planeri* (Table 2).

**Table 2:** Residues forming the LRRNT protrusion show limited sequence variability in VLRC molecules.

Genes	Sequence	Frequency (No. of sequences)
*VLRC (L. japonicum)*	NKTDSSPE	80.6% (83)
	SATTSSPE	18.4% (19)
	NKTESSPE	1.0% (1)
*VLRC (P. marinus)*	SATDSSPE	100% (60)
*VLRC (L. planeri)*	NKTESSPE	100% (2)

Flexibility in the LRRCT loop is important for VLRB molecules to bind antigens [20], [22]. However, the atomic b-factors in the LRRNT loop of the VLRC structure are around the same values as in other regions, suggesting that the flexibility in this region is not particularly high. This is probably because the loop is packed closely within adjoining molecules in the crystal lattice.

### Absence of a protrusion in the C-terminal cap

The LRRCT region in lamprey VLRA and VLRB molecules contains a stretch of amino acids that displays marked variations in length, amino acid composition and secondary structure [19]. This stretch of residues, known as a hypervariable insert, can form a loop structure or protrusion important for antigen recognition in both lamprey VLRA (VLRA.R2.1-HEL) [22]) and VLRB molecules (VLRB.RBC36-H-trisaccharide [19], VLRB.2D-HEL [20] and VLR4-BclA [21]) (Figure 2). In lamprey VLRC, the corresponding stretch is composed of only a few residues and shows limited diversity [13]. Therefore, it was predicted that, unlike VLRA or VLRB, VLRC would lack the capacity to form protrusions in the LRRCT [13]. Crystallographic analysis of VLRC confirmed this prediction (Figure 1).

## Discussion

The most striking observation made in this study is that the N-terminal cap of lamprey VLRC has a long loop protruding toward the concave surface (Figure 1). Importantly, the stretch of residues constituting the LRRNT protrusion is highly conserved in length and amino acid composition in lamprey VLRC molecules (Table 2 and Figure 5). Therefore, the protrusion in LRRNT appears to be a shared feature of all lamprey VLRC molecules. In contrast, sequence alignment suggests that all VLRA molecules and >90% of VLRB molecules lack the potential to form comparable protrusions in their N-terminal caps (Figure 5).

Among the members of the LRR family of proteins, platelet-receptor glycoprotein Ibα has a similar long protrusion in the LRRNT that extends toward the concave surface [40]. In Ibα, its ligand, von Willebrand factor, is recognized by both N- and C-terminal protrusions. Therefore, it is reasonable to postulate that the protrusion in LRRNT of lamprey VLRC may be involved in antigen recognition. In lamprey VLRC, neither the 5′-LRRCT nor the protrusion in LRRNT exhibits high levels of variability. Hence, as suggested earlier [13], the N- and C-terminal caps of VLRC might interact with conserved epitopes of restricted sets of antigen or invariant regions of molecules involved in antigen presentation.

In hagfish, only two types of VLRs, VLRA and VLRB, had been known until a third VLR was identified quite recently [25]. Detailed phylogenetic analysis of this newly identified VLR indicated that it is the counterpart of lamprey VLRA and that, in reality, the hagfish VLR molecule previously known as VLRA is the counterpart of lamprey VLRC, thus necessitating the change in nomenclature [25]. Consistent with this is the observation that, like lamprey VLRC, the hagfish VLRC (formerly known as VLRA) lacks a hypervariable insert, and hence a prominent protrusion in the LRRCT [20] (Figure 4). There are, however, some structural differences between lamprey and hagfish VLRC molecules. First, unlike lamprey VLRC, hagfish VLRC appears to lack the capacity to form a protrusion in the LRRNT (Figures 4 and 5). Second, unlike lamprey VLRC and other VLR molecules, hagfish VLRC has a 3_10_-helix upstream of the β1 strand. Whether these structural differences bear any functional significance is an issue that warrants further investigation.

Accumulating evidence indicates that the lymphocyte-based adaptive immune systems of gnathostomes and cyclostomes show remarkable similarity despite the fact that they use structurally unrelated molecules as antigen receptors [41]. Particularly striking is the occurrence of two major populations of agnathan lymphocytes, one involved in humoral immunity and another presumably involved in cell-mediated immunity. VLRB can be anchored to the cell membrane via a GPI linkage [4] or secreted like antibodies as pentamers or tetramers of dimers [15]. Polymerization of VLRB increases its avidity in a manner similar to multimer formation of IgM and IgA molecules. Overall, VLRB+ cells resemble B cells in that they both secrete antibodies in response to an antigen challenge. On the other hand, VLRA+ cells resemble T cells; not only do they undergo blastoid transformation in response to a T-cell mitogen, but they also express IL-17, GATA2/3 and NOTCH whose jawed vertebrate counterparts are expressed in T cells, and involved in their development and differentiation. The similarity between the adaptive immune systems of gnathostomes and cyclostomes was further bolstered by the recent proposal that VLRC+ cells constitute a second lineage of T-like cells and might be akin to γδ T cells [17]. In addition, a highly polymorphic hagfish membrane protein, NICIR3 (also called ALA), was identified as a predominant allogeneic leukocyte antigen recognized by VLRB antibodies [42], raising the possibility that NICIR3 might play a role comparable to that of MHC molecules of jawed vertebrates. It would be important to understand whether VLRC can recognize antigens directly like mammalian γδ TCRs or requires a functional MHC analog for antigen recognition.
